# Villitis of Unknown Etiology in the Placenta of a Pregnancy Complicated by COVID-19

**DOI:** 10.5146/tjpath.2020.01506

**Published:** 2021-05-15

**Authors:** Erdener Ozer, Erkan Caglıyan, Resmiye Irmak Yuzuguldu, Mustafa Cüneyt Cevizci, Nuray Duman

**Affiliations:** Department of Pathology, Dokuz Eylul University Hospital, Izmir, Turkey; Department of Obstetrics & Gynecology Dokuz Eylul University Hospital, Izmir, Turkey; Department of Neonatology, Dokuz Eylul University Hospital, Izmir, Turkey

**Keywords:** COVID-19, Placenta, SARS-CoV-2, Villitis of unknown etiology

## Abstract

Villitis of unknown etiology (VUE) is noninfectious chronic villitis thought to be associated with fetal growth restriction and stillbirth. COVID-19 and the pandemic SARS-CoV-2 infection can cause an increased risk in pregnant women for potential maternal and fetal complications from an immunological mechanism. We report a 39-week-gestational-age infant delivered to a 37-year-old mother diagnosed with SARS-CoV-2 infection at 37 weeks gestation. The placental examination showed the morphological features of VUE. We showed immunohistochemically that macrophages and CD4-positive T cells predominated in the villous tissue, although elevated numbers of CD8-positive cells were also present. We hypothesize that VUE may represent a maternal anti-viral immune response, in this case to SARS-CoV-2.

## INTRODUCTION

The emergence of the pandemic severe acute respiratory syndrome coronavirus 2 (SARS-CoV-2) infection and coronavirus disease (COVID-19) has created a serious public health problem. Covid-19 can affect pregnant women because they become an extremely sensitive group during any pandemic of viral infections due to altered immune functions and susceptibility to infection. Although Hosier et al. (1) has demonstrated SARS-CoV-2 to be localized to syncytiotrophoblast cells at the maternal-fetal interface of the placenta, there is no reliable evidence for vertical transmission of the virus in pregnant women with COVID-19; however, an increased prevalence of perinatal problems has been reported (2,3). It is also unexplained yet by which mechanism SARS-CoV-2 virus infection can be effective on the pregnancy outcome because there is little data on adverse pregnancy outcomes in pregnant women with COVID-19 (4).

Placental pathology can provide significant knowledge of SARS-CoV-2 virus infection in pregnancy regarding the wellbeing of both mother and fetus, thus regarding perinatal outcome. In a few recent cohort studies, the placentas of pregnant women infected with SARS-CoV-2 have shown higher rates of maternal or fetal vascular malperfusion features associated with adverse outcomes (5,6). We report herein a case of chronic villitis in the placenta of a pregnancy complicated by COVID-19. We hypothesize that chronic inflammatory or immune pathological lesions of the placenta may be also effective on the perinatal outcome in SARS-CoV-2 virus infection, and result from an anti-viral immune response.

## CASE REPORT

The patient was a 39-week-gestational-age infant delivered to a 37-year-old G2, P1 mother with no significant prior obstetric history. Three days prior to presentation, the mother developed fever, flu-like symptoms, mild sore throat, and a non-productive cough. The molecular detection test by reverse transcription–polymerase chain reaction (RT-PCR) for SARS-CoV-2 RNA in a nasopharyngeal swab obtained from the patient on admission was positive. She had no history of social contact with a COVID-19 positive individual. Neither her husband nor her older child had a positive test result for SARS-CoV-2. No further details were available to us.

The mother was admitted for labor and birth. Fetal ultrasound revealed a healthy fetus with an estimated fetal weight within expected range and normal amniotic fluid volume. Laboratory studies revealed normal liver transaminases, as well as normal partial thromboplastin time, fibrinogen, and D-dimer level. Blood smear revealed normal blood count with unremarkable morphology. She underwent cesarean section. The boy infant had a birth weight of 3360 grams and a good Apgar score. His RT-PCR test for SARS-CoV-2 was negative. Both mother and infant were discharged in good health.

Macroscopic and microscopic examinations of the placenta were performed according to the standard protocol. The placental disc measured 18x17x2.5 cm and weighed 564 g without the fetal membranes or umbilical cord (75th percentile). The umbilical cord showed three vessels and no thrombosis, was 14 cm in length, was inserted eccentrically, and measured 1.2 cm in diameter. Placental membranes were translucent, complete, and morphologically normal. The fetal surface showed subchorionic fibrin and both surfaces appeared otherwise normal. On cut section, there was no grossly visible villous lesion.

**Figure 1:**
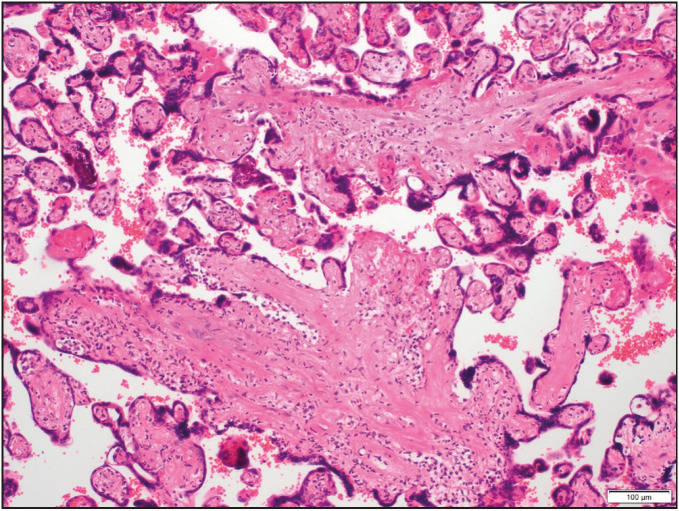
Low-grade chronic villitis characterized by a mononuclear cell infiltrate (<10 inflamed villi per focus) (H&E; x100).

**Figure 2:**
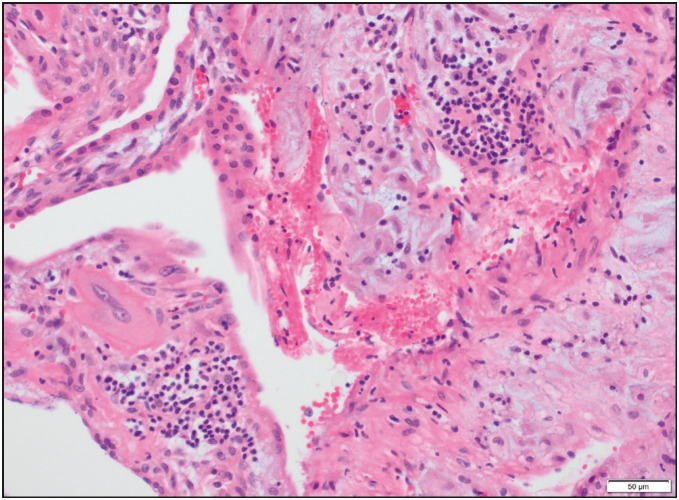
Chronic deciduitis: Predominantly lymphocytic infiltration in the decidua (H&E; x200).

**Figure 3:**
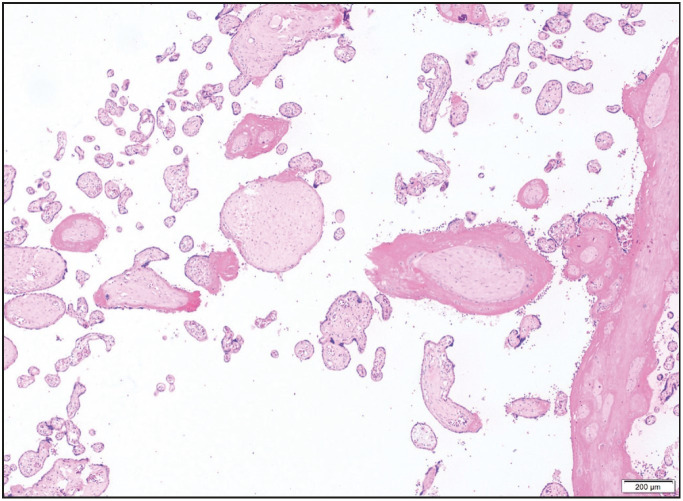
Avascular chorionic villi compatible with fetal vascular malperfusion (H&E; x40).

**Figure 4:**
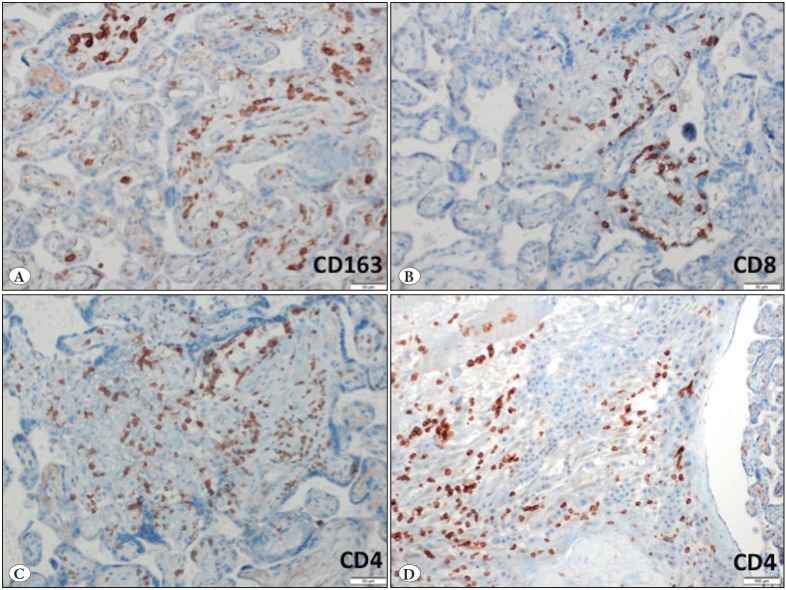
Immunohistochemical findings. **A)** CD163-positive macrophages in the villi (IHC; x200). **B)** CD8-positive T lymphocytes in the villi (IHC; x200). **C)** CD4-positive T lymphocytes in the villi (IHC; x200). **D)** CD4-positive T lymphocytes in the decidua (IHC; x100).

On histological examination, the villi showed focally an inflammatory infiltrate composed of macrophages and plasma cells as well as many lymphocytes (Figure 1). In addition, mononuclear cells in the decidua and avascular villi adjacent to a subchorionic thrombohematoma were present (Figure 2 Figure 3). The maternal vessels did not show any features of decidual vasculopathy. Neither histological chorioamnionitis nor funusitis were seen. The inflammatory infiltrate in the villi and decidua predominantly consisted of macrophages and CD4-positive T lymphocytes, as demonstrated by immunohistochemistry for CD4, CD8 and CD163 (Figure 4). The pathological diagnosis was made as low-grade chronic villitis graded according to the Amsterdam criteria (7). However, we were not able to demonstrate the presence of SARS-CoV-2 in the placental tissue with histological signs of chronic villitis, and therefore we classified the morphology as villitis of unknown etiology (VUE).

## DISCUSSION

The physiologic and immunologic changes that occur as a normal component of pregnancy can have systemic effects that increase the risk for complications from viral infections. We reported here a case of VUE in the placenta of a successful pregnancy complicated with SARS-CoV-2 infection.

The etiology of chronic villitis is not fully addressed yet. A large proportion of cases of chronic villitis is VUE and may reflect a noninfectious immune response (8,9). However, 41% of lesions originally classified as VUE have been reported in a study to have a viral infectious etiology demonstrated by electron microscopy (10). We did not plan to demonstrate the presence of SARS-CoV-2 because RT-PCR tests for SARS-CoV-2 RNA in the nasopharyngeal swab obtained from the infant were negative.

To the best of our knowledge, there is only one case reporting localization of SARS-CoV-2 in the placenta (1). However vertical transmission of the virus is still unproven. We do not know whether the clinical condition of the infected mother may be associated with the risk of viral transmission and the virus might also have been cleared from placental tissue depending on the time between viral infection and labor. We think that the chronic villitis in our case may be unrelated to viral transmission because the mother developed milder symptoms of COVID-19 and the symptoms appeared at the 39th week of pregnancy, only three days prior to presentation.

Recent data regarding VUE in live birth have shown that villous lesions are composed of mainly fetal macrophages and maternal CD4- or CD8-positive T lymphocytes (11). We showed immunohistochemically that macrophages and CD4-positive T cells predominated, although elevated numbers of CD8-positive cells were also present. This composition that we observed, with CD4 positive lymphocytes outnumbering CD8 positive lymphocytes, is in accordance with the study of Labarrere et al. (12) and supports the hypothesis that the anti-viral immune response in VUE is of the Th1-type including anti-fetal cell-mediated mechanisms.

There is an immunologic paradox in normal pregnancy, which is the shift from Th1- to Th2-type responses. Suppression of anti-fetal cell-mediated responses and a functional predominance of antibody-mediated immunity provide an advantage in the maintenance of a successful pregnancy. The cytokine profile in Th2-type responses includes mainly IL-4, IL-10, and TGF-β, whereas the anti-inflammatory cytokines IL-2, IL-6, and IL-12 predominate in Th1-type responses (13,14).

SARS-CoV-2-specific CD4+ T cell and antibody responses in lung lesions were observed in all COVID-19 cases, whereas CD8+ T cell responses were observed in not all but most patients in a study (15). In addition, the immunological reaction triggered by SARS-CoV- 2 infection has been shown to be effective in mobilizing numerous cytokines such as IL-1, IL-6, IL-12, IFN-γ, and TNF-α, preferentially targeting lung tissue (16). Therefore, we think that there may be a strong similarity in the characteristics of inflammatory cell distribution and cytokine production among the lung lesions and placental VUE lesions of COVID-19 patients. In accordance with our suggestion, increased levels of IL-2 and IL-12 have been shown in VUE lesions (11). This likely indicates that there may be an inappropriate shift toward the Th2-type immune response in pregnant women with VUE lesions, which may explain the higher risk of stillbirth. Therefore, the presence of VUE may carry a risk of adverse perinatal outcome for COVID-19 infected pregnant patients.

In a recent cohort study, maternal Covid-19 infection was found to be significantly associated with fetal vascular thrombosis (5). Besides, VUE sometimes progresses to obliterative fetal vasculopathy. The presence of cytotoxic CD8-positive T cells is responsible for the development of the fetal placental vasculopathy often observed in VUE and also the increased apoptosis of intravillous cells (9,17). These may explain why we did not find any features of fetal vascular malperfusion in our case because CD8-positive lymphocytes were relatively low in the lesions. In addition, obliterative fetal vasculopathy occurs in response to chronic inflammation in VUE when there is multifocal involvement (9). This may also explain the absence of fetal vascular malperfusion because chronic villitis in our case was of low grade (<10 inflamed villi per focus). Furthermore, there was no gross umbilical cord abnormality or maternal hypercoagulability known to be associated with fetal vascular malperfusion.

We conclude that SARS-CoV-2 infection may be related to VUE resulting from an anti-viral response. As SARS-CoV-2 is a virus, it is likely to induce inflammation. This is a Th1-type response including several types of T cells, predominantly CD4-positive lymphocytes resulting from a disturbance of normal pregnancy tolerance. The immunogenic activity of these cells is influenced by cytokines that are increased in SARS-CoV-2 infection as well. We therefore hypothesize that the burst of inflammatory cytokines accompanying SARS-CoV-2 infection may produce lesions of VUE. Further studies are needed to prove this hypothesis regarding whether VUE is related to an antiviral immune response in COVID-19.
